# Breast radiotherapy planning: A decision‐making framework using deep learning

**DOI:** 10.1002/mp.17527

**Published:** 2024-12-03

**Authors:** Pedro Gallego, Eva Ambroa, Jaime PérezAlija, Nuria Jornet, Cristina Anson, Natalia Tejedor, Helena Vivancos, Agust Ruiz, Marta Barceló, Alejandro Dominguez, Victor Riu, Javier Roda, Pablo Carrasco, Simone Balocco, Oliver Díaz

**Affiliations:** ^1^ Radiofísica i Radioprotecció Hospital de la Santa Creu i Sant Pau Barcelona Spain; ^2^ Department de Matemàtiques I Informàtica Universitat de Barcelona Barcelona Spain; ^3^ Medical Physics Unit, Radiation Oncology Department Consorci Sanitari de Terrassa Terrassa Spain; ^4^ Computer vision Center Bellaterra Spain

**Keywords:** 3D‐CRT, breast planning, IMRT

## Abstract

**Background:**

Effective breast cancer treatment planning requires balancing tumor control while minimizing radiation exposure to healthy tissues. Choosing between intensity‐modulated radiation therapy (IMRT) and three‐dimensional conformal radiation therapy (3D‐CRT) remains pivotal, influenced by patient anatomy and dosimetric constraints.

**Purpose:**

This study aims to develop a decision‐making framework utilizing deep learning to predict dose distributions, aiding in the selection of optimal treatment techniques.

**Methods:**

A 2D U‐Net convolutional neural network (CNN) model was used to predict dose distribution maps and dose‐volume histogram (DVH) metrics for breast cancer patients undergoing IMRT and 3D‐CRT. The model was trained and fine‐tuned using retrospective datasets from two medical centers, accounting for variations in CT systems, dosimetric protocols, and clinical practices, over 346 patients. An additional 30 consecutive patients were selected for external validation, where both 3D‐CRT and IMRT plans were manually created. To show the potential of the approach, an independent medical physicist evaluated both dosimetric plans and selected the most appropriate one based on applicable clinical criteria. Confusion matrices were used to compare the decisions of the independent observer with the historical decision and the proposed decision‐making framework.

**Results:**

Evaluation metrics, including dice similarity coefficients (DSC) and DVH analyses, demonstrated high concordance between predicted and clinical dose distribution for both IMRT and 3D‐CRT techniques, especially for organs at risk (OARs). The decision‐making framework demonstrated high accuracy (90%), recall (95.7%), and precision (91.7%) when compared to independent clinical evaluations, while the historical decision‐making had lower accuracy (50%), recall (47.8%), and precision (78.6%).

**Conclusions:**

The proposed decision‐making model accurately predicts dose distributions for both 3D‐CRT and IMRT, ensuring reliable OAR dose estimation. This decision‐making framework significantly outperforms historical decision‐making, demonstrating higher accuracy, recall, and precision.

## 1 INTRODUCTION

Breast cancer is the first most common cancer in the world, representing about 12% percent of all cancers.^1^ According to the World Health Organization, around 2.3 million women are diagnosed per year, resulting in 685.000 deaths worldwide. Thus, the significant prevalence and impact of breast cancer contribute substantially to the economic burden on health care systems.

Once breast cancer is detected, treatment options vary between individuals but could include surgery, chemotherapy, hormone therapy, targeted therapy, immunotherapy, and/or radiotherapy. The use of radiation therapy has experienced a significant rise in breast cancer treatment over the last three decades despite variations in its application worldwide.^1^

To ensure a patient's long‐term survival and good quality of life after radiotherapy, it is important to personalize the optimal irradiation of the tumor (i.e., target volume), maintaining low doses to the surrounding tissues and organs, which could otherwise cause toxicity or even mortality.^2^

Since 2000, the use of inverse planning techniques, such as intensity‐modulated radiation therapy (IMRT), has increased significantly.^3^ Several studies compared the capability of three‐dimensional conformal radiation therapy (3D‐CRT) and intensity‐modulated techniques for breast cancer treatment planning.^4^, ^5^, ^6^ On one hand, IMRT usually achieves better planning target volume (PTV) coverage and lower organ at risk (OAR) doses, leading to a more conformal dose distribution than 3D‐CRT plans. On the other hand, intensity‐modulated techniques frequently increase the lower doses administered to the entire patient, increase delivery time, and could be a less robust treatment. The choice between planning techniques usually involves balancing the need to achieve planning dose constraints and the need to keep the approach as simple as possible. Therefore, IMRT is commonly used in cases of complex geometries when the prescribed dose constraints are hard to achieve, while 3D‐CRT is reserved for patients with anatomies that can meet the prescribed dose constraints.

The process of treatment planning is inherently time‐consuming, regardless of the chosen technique. The proficiency and experience of the clinical user (i.e., radiation therapist (RTT), medical physicist (MP), and radiation oncologist (RO)) may influence the quality of the treatment plan. While existing knowledge‐based planning tools have facilitated centers in attaining greater uniformity, efficiency, and quality in treatment plans,^7^, ^8^, ^9^ as of present, no tool exists to preemptively determine the optimal breast radiotherapy planning technique choice for a specific patient. The absence of such a tool often leads to an overuse of IMRT for a variety of reasons, including the relative ease of creating an IMRT plan.

During the last decade, deep learning has yielded substantial improvements in image processing and computer vision‐related tasks. Such technology has been proven to be a powerful tool to predict dose distributions and dose‐volume histograms (DVH) for various cancer treatment.^10^, ^11^, ^12^, ^13^

In this context, the convolutional neural network (CNN) U‐Net architecture^14^ has significantly enhanced the ability to predict radiation dose distributions of the patient, which can lead to reducing the operator's dependency and potential introduction of biases (i.e., manual creation of the treatment plan). This predicted dose distribution provides valuable visual input and DVH metrics, aiding MPs, RTTs and ROs in their initial decision‐making process to establish both technique and more attainable planning objectives before the formal planning stage.

Recently, authors have undertaken the prediction of dose distributions for breast treatments^15^, ^16^ using U‐Net‐type networks, but so far, they have mainly focused on IMRT, and therefore, it cannot be used as a support for treatment decision making process.

To train models for both 3D‐CRT and IMRT, one must consider that selecting patients in a clinical workflow where both techniques are being used routinely may introduce a selection bias.^17^ This is because more complex anatomies are likely to be treated with IMRT, while simpler ones are treated with 3D‐CRT. For this reason, patient selection must be carefully considered.

In the complex scenario of breast cancer treatment planning, establishing the most appropriate treatment planning technique presents a challenging task that has not yet been overcome. Therefore, in this article, we propose a decision‐making framework based on deep learning to predict dose distributions for both IMRT and 3D‐CRT treatment plans. This model thereby facilitates the assessment of DVHs and allows the less biased selection of the optimal treatment technique for each individual.

Such a tool allows us to determine whether all dose constraints can be met before creating a treatment plan, minimizing inter‐observer variability and saving effort and time during the planning treatment process. Moreover, this approach is a novel method for treatment planning decision‐making using U‐Net dose prediction in radiotherapy.

This study aligns with the American Society for Radiation Oncology (ASTRO)'s^18^ campaign, which recommends judicious use of IMRT over 3D‐CRT only when significant clinical benefits are expected.

## 2 METHODS

The methodology proposed in this paper follows international recommendations to ensure reproducibility of machine learning algorithms, that is, the Checklist for Artificial Intelligence in Medical Imaging (CLAIM),^19^ and the AAPM task group report 273.^20^

### 2.1 Dataset

The intended sample size for this study was determined based on several considerations to ensure robustness and generalizability of the deep learning models developed. Initially, retrospective datasets from two medical centers, Hospital Plató (${\mathrm{Inst}}_{1}$) and Hospital de la Santa Creu i Sant Pau (${\mathrm{Inst}}_{2}$), were selected to encompass a diverse range of patient anatomies, dosimetric protocols, and clinical practices associated with breast cancer treatment planning using both 3D‐CRT and IMRT techniques.

In ${\mathrm{Inst}}_{2}$, both 3D‐CRT and IMRT techniques were used for breast treatments. However, the clinical criteria were to use 3D‐CRT as the default approach, and only in the cases where clinical constraints could not be adequately fulfilled with 3D‐CRT should IMRT be chosen. Therefore, a model trained exclusively on this database would be biased towards that selection criteria.

${\mathrm{Inst}}_{1}$ did not use IMRT for breast treatments. Consequently, all breast patients, regardless of their geometry and the challenges in fulfilling dose constraints, were treated using 3D‐CRT, so ${\mathrm{Inst}}_{1}$ is not affected by selection bias.

Patient inclusion criteria encompass left and right breasts with or without the involvement of axillary and supraclavicular lymph nodes, while exclusion criteria encompass those with internal mammary nodal chain involvement and boost. All treatment plans were renormalized to 2 Gy/fraction, in 25 fractions, to consider different prescribed doses. Local ethical committee approval (EC/23/332/7438 RFRp001) was granted for this study, and all personal data were anonymized using the Dicompyler anonymization tool.^21^

From ${\mathrm{Inst}}_{1}$, 204 consecutive patients who met the inclusion criteria from 2015 to 2017 were selected. All computed tomography (CT) images were acquired from the same scan, a Brighspeed (General Electric Healthcare, Chicago, IL, USA) with a 512 pixels × 512 pixels resolution and a slice thickness of 3.75 mm. The planning was performed using Eclipse treatment planning system (TPS) v15.1, with the Anisotropic Analytical Algorithm (AAA) algorithm, and delivered with Varian Clinac 2100 C/D and Varian Clinac 600 (Varian Medical Systems, Palo Alto, CA, USA), both with Millenium 120 multileaf collimator.

In the case of ${\mathrm{Inst}}_{2}$, 52 3D‐CRT consecutive patients and 90 IMRT consecutive patients from years 2018 to 2022 who met the inclusion criteria were selected. All CT images were acquired from a Brilliance Big Bore scan (Philips, Andover, MA, USA) with a resolution of 512 pixels × 512 pixels and a slice thickness of 3 mm. The plan was performed using the Eclipse TPS v15.1, with the AAA algorithm, and delivered with Varian Clinac 2100 C/D and Varian TrueBeam (Varian Medical Systems, Palo Alto, CA, USA), both with Millenium 120 multileaf collimator.

The number of patients selected for each group, along with their detailed characteristics, is depicted in Table 1.

**TABLE 1:** Patient characteristics in this study.

Dataset	Number of patients	IMRT	3DCRT	Average age	Breast PTV	Lymph nodes PTV	Left breast	Right breast
Institution 1	204	0	204	57 ± 13	204	153	100	104
Institution 2	142	90	52	65 ± 14	142	51	73	69

Abbreviations:3DCRT, three‐dimensional conformal radiation therapy; IMRT, intensity‐modulated radiation therapy; PTV, planning target volume.

All OAR volumetric delineations were carried out by experienced RTTs under the supervision of the RO assigned to the respective patient as part of the clinical workflow using the Eclipse TPS (Varian Medical Systems, Palo Alto, CA, USA). The assigned RO delineated the patients' PTVs and underwent a peer review process before treatment to ensure minimal variability between ROs.

${\mathrm{Inst}}_{1}$ followed the Radiation Therapy Oncology Group (RTOG) Breast Cancer Atlas, while ${\mathrm{Inst}}_{2}$ adhered to the contouring guidelines recommended by the European Society for Radiotherapy and Oncology (ESTRO).^22^

### 2.2 Deep learning model

To predict dose distributions, we used a 2D U‐net^14^ Xception‐style model,^23^ which is a well‐established architecture used in the field of radiation therapy for dose prediction.^24^, ^25^, ^26^, ^27^ This CNN receives as input a 2D CT slice and its corresponding 2D binary mask obtained from a 3D manual volumetric delineations of the following regions of interest, all done inside Eclipse TPS. For the PTV, breast PTV and lymph node PTV (if applicable) were used, whereas for the OARs, heart, lung, and body were used. The U‐NET outputs the 2D dose matrix associated with the input 2D CT slice and its corresponding mask.

In this study, we chose a 2D U‐Net over a 3D network due to several advantages. While 3D networks can capture more spatial information by processing entire volumes, they require significantly more data and computational power, making them prone to overfitting and instability, especially with smaller datasets.^28^ In contrast, 2D networks like the 2D U‐Net are more robust with limited data, as they can be trained on individual slices, increasing the number of training samples and often leading to better performance in such contexts.

### 2.3 Data preparation

**FIGURE 1:**
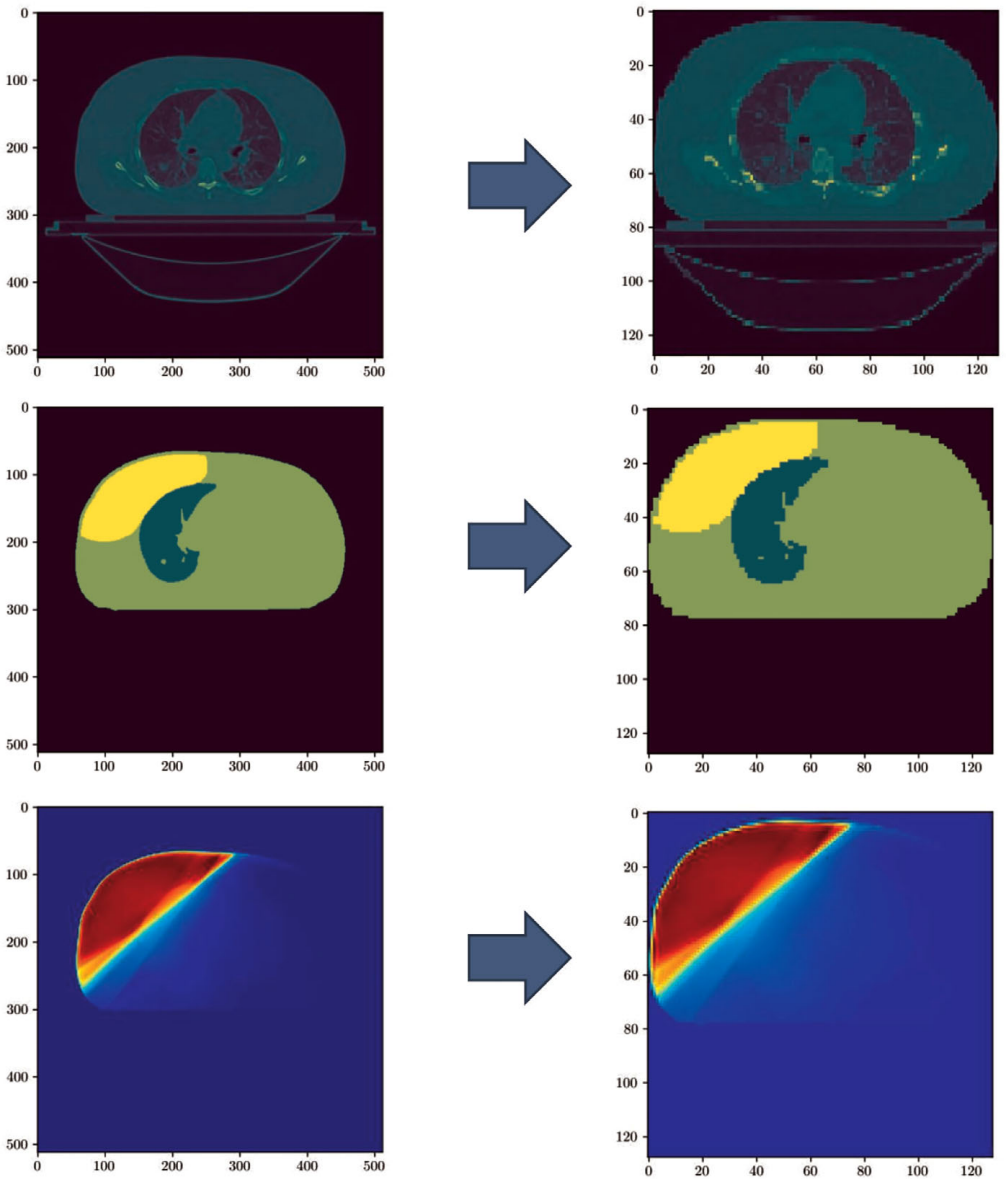
Examples of input and output of the U‐NET. CT Image (top) and mask (middle) of breast, lung and body represent the input of the network whereas dose matrix (bottom) corresponds to the output. Left and right column illustrates the original and the modified version (resampled and croped) for each case. Different color maps have been used for visualization purposes. CT, computer tomography.

Each 2D CT slice, along with the corresponding mask and dose distribution, underwent cropping procedures to optimally fit the body structure within the imaging frame, as shown in Figure 1.

Subsequently, the input and output data for training were uniformly re‐sampled, using the nearest neighbor re‐sampling method, to achieve an image dimension of 128 × 128 pixels.

### 2.4 Training

**FIGURE 2:**
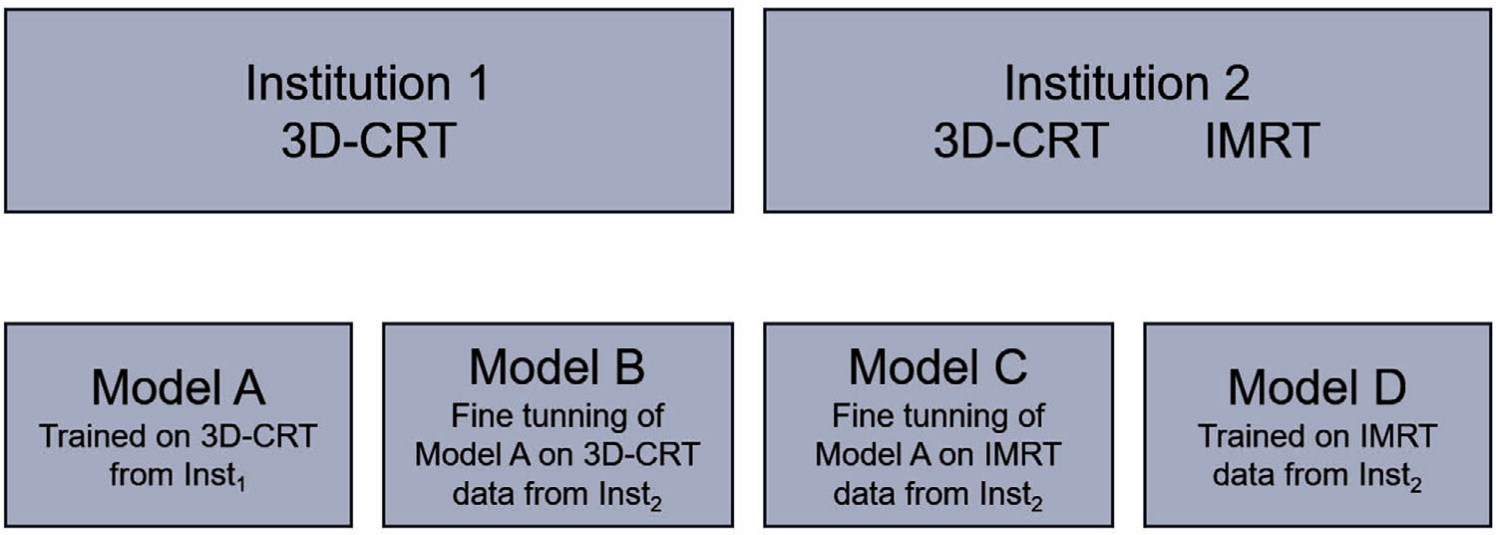
Summary of models developed in this study.

Four different U‐Net models ($Model_{x}$) were trained as described below in Figure 2.

- $Model_{A}$: A model trained from scratch with data from ${\mathrm{Inst}}_{1}$ (only 3D‐CRT patients). A k‐fold cross‐validation (k = 7) was used to train this model. Each fold sequentially served as the test set, while the remaining folds were used for training. This iterative process ensured that all patients from ${\mathrm{Inst}}_{1}$ were eventually included in the test set across different models.
- $Model_{B}$: Given that the patients from $Inst_{1}$ and $Inst_{2}$ were treated using different CT systems, diverse dosimetric protocols, and different medical experts contouring, to apply the $Model_{A}$ (trained on $Inst_{1}$ data only) to $Inst_{2}$ data, a fine‐tuning process was performed (only 3D‐CRT patients). All layers were retrained during this fine‐tuning process to ensure the model adapted effectively to the new dataset. Such a strategy was developed to mitigate the previously mentioned treatment selection bias. For this model, k‐fold cross‐validation (k = 7) was used.
- $Model_{C}$: A fine‐tuning of $Model_{A}$ using the IMRT patients from ${\mathrm{Inst}}_{2}$. For this model, k‐fold cross‐validation (k = 7) was used. All parameters of the model are initialized equal to $Model_{A}$ and fine‐tuned in this process.
- $Model_{D}$: A model trained from scratch with the IMRT patients from ${\mathrm{Inst}}_{2}$. This was carried out to verify whether the strategy of fine‐tuning $Model_{A}$ was an effective bias mitigation approach. For this model, k‐fold cross‐validation (k = 7) was used.

All models were implemented using the high‐level Keras API^29^ embedded in TensorFlow.^30^ All computations were undertaken on a personal computer with an Intel Core i7 processor and a GeForce RTX 1070 Ti graphics card. In all cases we have used random parameter initialization, a mean square error as a loss function (voxels‐wise difference in dose distributions), 20 epochs, and ADAM as our optimizer with the following empirically chosen parameters: a learning rate of 0.001, $beta_{1}=0.9$, $beta_{2}=0.999$.

### 2.5 Model evaluation

The predicted dose distribution map was compared against the reference dose distribution for all patients used in the model for all four models. Dice Similarity Coefficients (DSC) of the prescribed dose were computed (between 0% and 100%) to quantitatively assess the concordance of spatial dose distributions between the clinical and predicted dose profiles, similarly as described by Kandalan et al.^31^

In addition to the dose distribution, we generated a DVH for each patient and we compared it with the DVH obtained clinically. From the DVH, the following metrics were computed: V95% and mean dose for the PTV Breast and Lymph Nodes PTV, if present, V25 Gy and mean dose for the Heart, and V20 Gy and mean dose for the ipsilateral Lung as this were the clinical constraints followed. For the comparison of dose DVH variables, we used the following metric:

$$\frac{D_{Clinical}-D_{Predicted}}{D_{Presciption}}\times 100\%,$$

where $D_{Clinical}$ is the corresponding clinical DVH metric selected, $D_{Predicted}$ is the prediction for this DVH metric and $D_{Presciption}$ is the prescription target dose for each patient. For volume metrics, such as V20 Gy and V25 Gy, the comparison was performed directly by calculating the difference between the clinical and predicted values, without normalization.

### 2.6 Feasibility study of a decision‐making framework

We selected a cohort of 30 consecutive patients from $Inst_{2}$, from January 2023 to March 2023, not included in the original databases used in training, to perform an external validation of the proposed framework. This cohort was divided equally between those planned using IMRT (15) and those planned using 3D‐CRT (15). Each patient underwent both IMRT and 3D‐CRT new planning, performed manually by an experienced dosimetrist. An independent MP, blinded to the planning technique, evaluated the plans and selected the optimal one based on predefined clinical criteria: V20 Gy < 30% for the ipsilateral lung, V25 Gy<10% and mean heart dose < 3 Gy, along with metrics related to the PTV.

For the 3D‐CRT plans, we used tangential fields with wedges and up to three field‐in‐field segments to achieve optimal dose distribution. In the IMRT plans, we employed three beams per tangential incidence, each separated by 10 degrees. Additional fields were added if the predefined constraints were not met, adjusting based on the patient’s geometry. All plans were developed following $Inst_{2}$’s protocols to ensure they were both realistic and feasible for delivery.

To validate the proposed decision‐making framework, we restricted our analysis to data from $Inst_{2}$, as this is the only institution employing both 3D‐CRT and IMRT techniques. Therefore, for 3D‐CRT only $Model_{B}$ is applicable. For IMRT, the choice between $Model_{C}$ and $Model_{D}$ will be based on the comparative performance of $Model_{C}$ versus $Model_{D}$ regarding OARs. These models were used to predict treatment plans for each patient for both techniques. The decision‐making framework applied our clinical constraints regarding OARs (V20 Gy < 30% for the ipsilateral lung, V25 Gy < 10%, and mean heart dose < 3 Gy) to determine the recommended treatment technique.The protocol employed used 3D‐CRT by default if the plan met the constraints, and opted for IMRT only if 3D‐CRT failed to meet the constraints that IMRT could satisfy.

To maximize the use of all available training patients, both models were retrained with the full set of training patients available for each case, following the same methodology outlined in training section.

With these predictions, two confusion matrices were generated. The first matrix compared the decisions made by the independent observer, who had access to both manually created plans (considered our ground truth) against the clinical decisions recorded within the patient’s clinical history, often based on the judgment of the RTT or MP handling the case. The second matrix compared ground truth against the recommendations of the proposed decision‐making framework. For each confusion matrix, accuracy, recall, precision, and F1 score were calculated.

## 3 RESULTS

**FIGURE 3:**
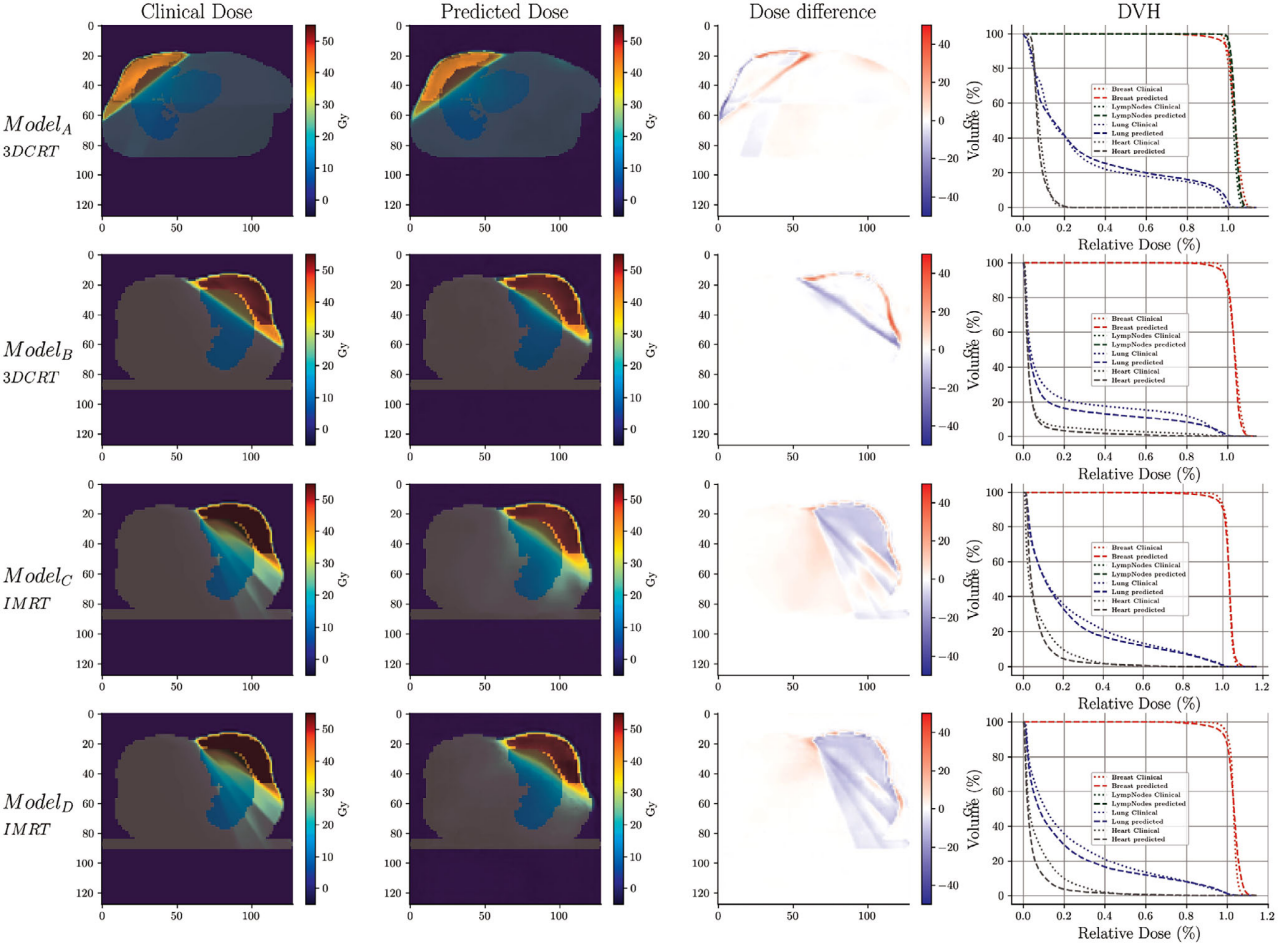
Dose comparison between the clinical plan and the predicted plan using the four models trained, the absolute dose difference, and the DVH comparisons. For $Model_{A}$, a test set patient from Institution 1 was used, while $Model_{B}$, $Model_{C}$, and $Model_{D}$ utilized a test set patient from Institution 2. DVH, dose‐volume histogram.

Figure 3 presents an example of the predicted dose distribution for random patients used in each model, compared to the clinical dose received, for each of the four models ($Model_{x}$). It is observed that the estimated distributions (second column) are similar to the clinical reference (first column), and the differences are primarily located in the peripheral regions (see third column) of the PTV. Similar correspondence can be observed in the DVH curves in each of the four models (fourth column). Additionally, this picture also illustrates that the discrepancies in the IMRT models, both with fine‐tuning and from scratch (rows 3 and 4 of Figure 3), are slightly larger than in the 3D‐CRT models.

**FIGURE 4:**
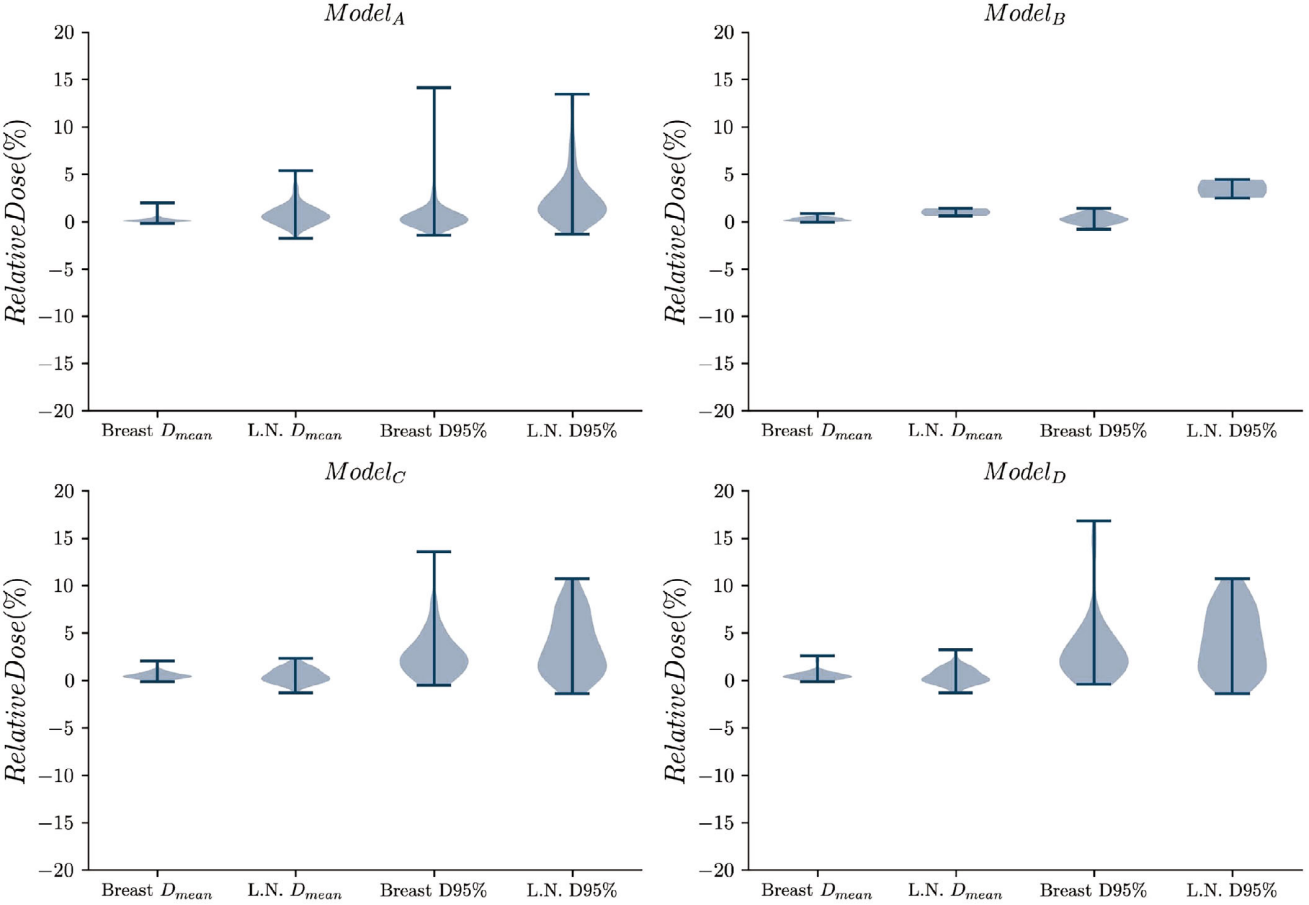
Violin plot of the relative differences (in %) found between the clinical and predicted plan for the PTV metrics for each of the models investigated ($Model_{X}$). PTV, planning target volume.

**FIGURE 5:**
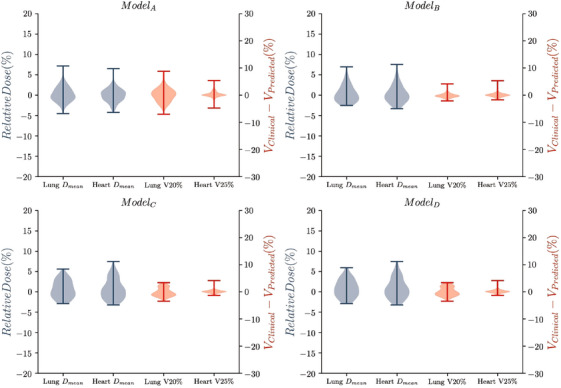
Violin plot of the relative differences (in %) found between the clinical and predicted plan for the OAR metrics for each of the models investigated ($Model_{X}$).The blue scale corresponds to the differences of $D_{mean}$ metrics, while the red scale corresponds to the differences in Lung V20% and Heart V25% metrics. OAR, organ at risk.

In Figures 4 and 5, a comparison between the selected dosimetric values between the prediction and the reference is shown in a violin plot. For the metrics related to the PTV, the largest differences are observed in the distributions of the PTV D95% for the $Model_{A}$ and both IMRT models ($Model_{C}$ and $Model_{D}$). However, $Model_{B}$ proves to be more accurate, except for the lymph nodes D95% (Table 2). For the OAR metrics, the distributions obtained are nearly centered around 0, with standard deviations from 1.5% to 5% as shown in Table 2.

**TABLE 2:** Average differences in PTV and OAR metrics, along with one standard deviation, were calculated between the clinical and predicted plans for each model.

		Differences (%)			
ROI	Metric	Model_A	Model_B	Model_C	Model_D
Breast PTV	D_mean	0.3 ± 0.5	0.5 ± 0.4	1.0 ± 0.7	1.1 ± 1.0
	D95%	1.3 ± 3.5	0.6 ± 1.0	5.5 ± 4.9	6.6 ± 6.9
Lymph nodes PTV	D_mean	1.5 ± 2.2	2.0 ± 0.8	1.0 ± 1.5	0.9 ± 1.7
	D95%	5.0 ± 5.7	6.9 ± 1.9	7.4 ± 6.3	8.2 ± 6.7
Heart	D_mean	0.08 ± 3.48	0.6 ± 4.5	1.3 ± 4.9	1.6 ± 4.9
	V25Gy	0.2 ± 1.6	0.2 ± 1.4	0.3 ± 1.3	0.4 ± 1.3
Lung	D_mean	0.4 ± 3.8	0.7 ± 4.2	1.2 ± 4.3	1.5 ± 4.3
	V20Gy	0.2 ± 3.6	−0.2 ± 1.3	−0.2 ± 2.0	0.1 ± 2.1

Abbreviations: OAR, organ at risk; PTV, planning target volume.

**FIGURE 6:**
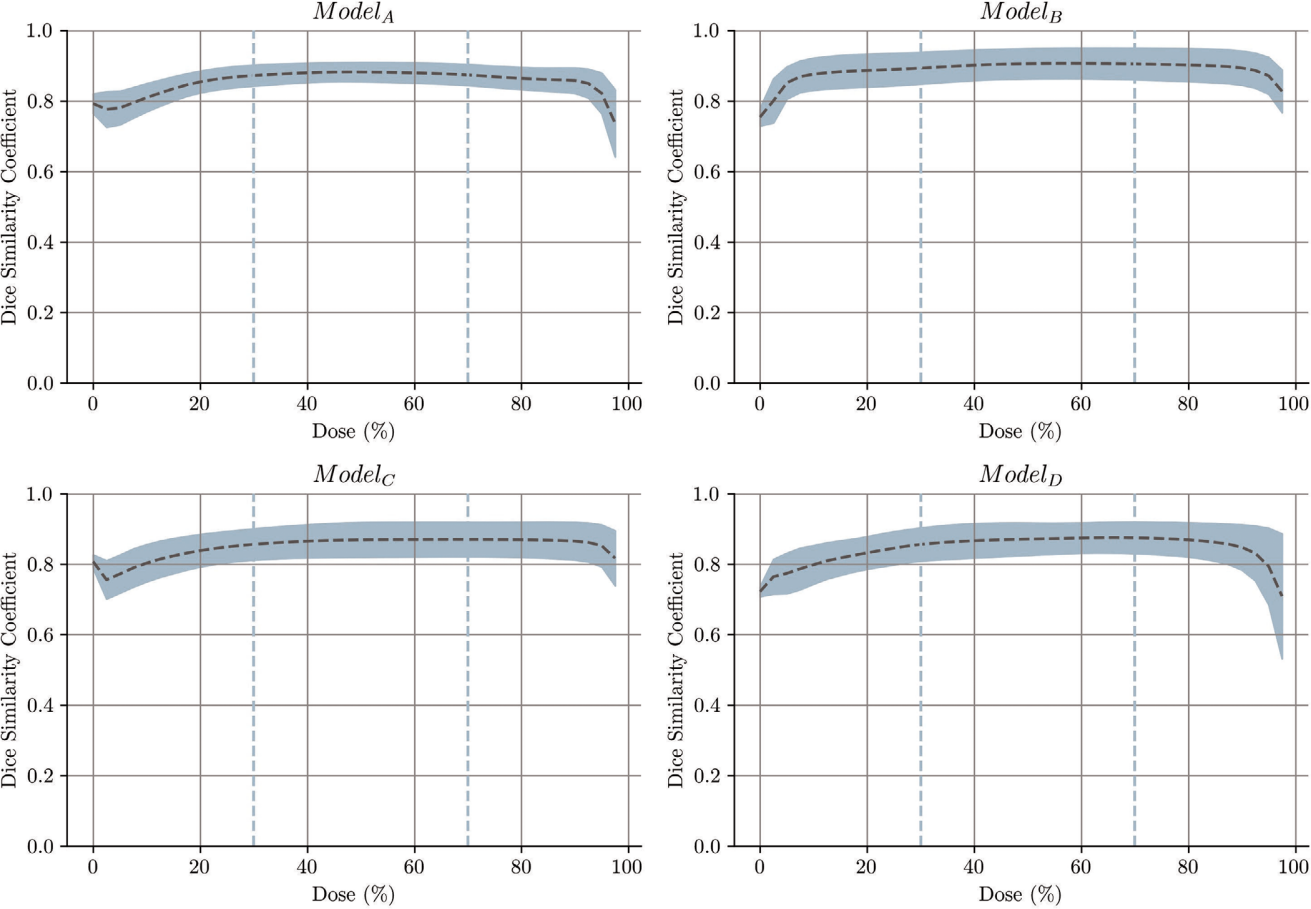
Analysis of DSC involved comparing isodose volumes between the clinically planned dose and the predicted dose. The graphs depict errors with one standard deviation, and the 30%‐70% range is demarcated by vertical dotted lines in the figure. DSC, dice similarity coefficients.

The DSC values across isodose volumes between clinical and predicted doses are depicted in Figure 6 for each of the trained models. It can be observed that, overall, high prediction performances (above 80% in most of the cases) are achieved across all ranges, with particularly good results in the mid to high range, which are areas of heightened significance. Comparing $Model_{C}$ with $Model_{D}$ shows better agreement for the first model, especially at doses close to 100%.

To test the decision‐making framework, $Model_{B}$ and $Model_{C}$ were used, with the latter chosen due to its better performance compared to $Model_{D}$ (Table 2, Figure 6).In comparing the historical decision and the decision‐making framework, as shown in Table 3, the results exhibit a mix of true positives, true negatives, false positives, and false negatives. The recall rate for this comparison was 47.8%, precision was 78.6%, accuracy was 50%, and the F1 score was 59.5%.

**TABLE 3:** Confusion matrix between the decisions made by the independent observer (ground truth) against the clinical decisions made historically.

	Actually 3D‐CRT	Actually IMRT
Historical 3D‐CRT	11	3
Historical IMRT	12	4

Abbreviations: 3DCRT, three‐dimensional conformal radiation therapy; IMRT, intensity‐modulated radiation therapy.

In the second comparison between the independent observer's decision (ground truth) and the decision‐making framework, represented in Table 4, the outcomes were more favorable. The recall rate in this comparison was 95.7%, precision was 91.7%, accuracy was 90.0%, and the F1 score was 93.6%.

**TABLE 4:** Confusion matrix between the decisions made by the independent observer (ground truth) against the decision making framework.

	Actually 3D‐CRT	Actually IMRT
Predicted 3D‐CRT	22	2
Predicted IMRT	1	5

Abbreviations: 3DCRT, three‐dimensional conformal radiation therapy; IMRT, intensity‐modulated radiation therapy.

## 4 DISCUSSION

In this study, we developed a CNN‐based deep learning architecture to predict dose distributions for both 3D‐CRT and IMRT in breast cancer treatment cases. As stated in the introduction, our main goal is to provide a decision‐making framework that aids healthcare professionals in selecting the most appropriate treatment technique based on predicted dose distributions from CT images of the patients.

The motivation for developing a decision‐making framework between IMRT and 3D‐CRT in our study is aligned with the recommendations of the ASTRO in its *Choosing Wisely* campaign. ASTRO advises against the routine use of IMRT for whole‐breast irradiation unless significant advantages in dose reduction to critical organs are anticipated. While IMRT can offer more precise dose distribution, these benefits do not always translate into significant clinical advantages, making 3D‐CRT a viable and often preferable option.

Our approach leverages deep learning techniques, specifically a 2D U‐net model, to predict dose distributions for both IMRT and 3D‐CRT. The utilization of the U‐net architecture, initially designed for biomedical image segmentation, facilitates accurate dose predictions without the need of manual treatment planning (which is very time‐consuming). Such a model not only supports the visualization of organ dose distributions but also provides DVH metrics, aiding physicians in decision‐making before formal planning.

The common clinical practice of using IMRT for complex anatomies and employing 3D‐CRT for simple cases introduces a potential selection bias. To address this issue, we selected patients from two institutions, trained models with patients from one of them ($Inst_{1}$), and fine‐tuned the model to adapt it to a different institution($Inst_{2}$).

To further ensure the generalization capabilities of our dataset and consequently the model, patients from $Inst_{1}$ exclusively underwent treatment using 3D‐CRT. This deliberate choice ensures that the database remains free from the aforementioned bias, irrespective of the anatomical complexity of the cases included.

The developed models demonstrated promising results in predicting dose distributions for 3D‐CRT. The quantitative and qualitative analysis, including DSC values greater than 0.8 in all cases, indicated a high concordance between predicted and clinical dose profiles. The presented histograms illustrating DVH metrics for PTV and OARs further validated the reliability of our predictions.

It is interesting to note that IMRT models, that is, $Model_{C}$ and $Model_{D}$, exhibit slightly inferior performance in PTV‐related metrics compared to other models. However, this marginal difference in PTV prediction accuracy does not significantly impact the overall utility of the models in aiding treatment technique decisions, as the primary concern lies in the accurate prediction of OAR metrics. $Models_{C}$ and $Models_{D}$ demonstrate strong predictive capabilities in OAR‐related metrics, thus emphasizing their relevance and effectiveness in this critical area of interest, with $Model_{C}$ showing slightly better predictions. Hence, we can conclude that in $Inst_{2}$, the application of $Model_{B}$ and $Model_{C}$ enables reliable estimation of OAR doses for each technique, and therefore, we can use $Model_{B}$ and $Model_{C}$ to select the most suitable technique for individual patients based on OAR doses. Additionally, for $Inst_{1}$, $Model_{A}$ demonstrates favorable results and can be an effective tool for patient‐specific quality assurance. However, since $Inst_{1}$ does not utilize IMRT, no decision‐making is required in this context.

The results of our study reveal notable differences in the performance of the decision‐making framework when compared to historical decisions and those made by an independent observer. As evidenced in Table 3, the historical decisions yielded a modest performance compared with the ground truth, with an accuracy of 50%, a recall of 47.8%, and an F1 score of 59.5%. These metrics indicate a considerable number of false negatives (12) and a moderate level of false positives (3), suggesting that the historical decision process is not very accurate at choosing the right technique. Indeed, it can be observed that, in general, more IMRT was administered than necessary.

Instead, the decision‐making framework, as detailed in Table 4, showed better results, achieving a recall, precision, accuracy, and F1 score superior to 90%. These improvements highlight the effectiveness of the framework in minimizing false negatives (1) and maintaining a low number of false positives (2), thereby providing a more reliable and consistent decision‐making process than manual decisions.

We did not evaluate low doses in this study, as these were not part of the routine clinical assessments in the institutions involved. However, in centers where low‐dose evaluation is critical, this could pose a problem. As seen in Figure 3, the IMRT models tend to have higher prediction errors in the low dose regions. This suggests that in institutions where low doses are closely monitored, such as V5Gy, a more careful approach and additional actions may be necessary to ensure that the predictions meet the clinical plans.

One limitation of the model is that it focuses solely on 3D‐CRT and IMRT without incorporating alternative treatment modalities such as volumetric arc therapy (VMAT) or proton therapy (not available). Although VMAT is not currently used for breast treatment in both institutions, it could be considered in future research as it may offer advantages in specific clinical scenarios where faster treatment delivery is beneficial, for example, in breath‐hold techniques where reducing the duration of each treatment session can improve patient comfort and reduce the challenges associated with maintaining a stable breath‐hold. Thus, to further validate the robustness of the model, a multi‐centric study with those techniques would be desirable. In such cases, the model evaluation would likely require a fine‐tuning process with other institution data to optimize performance and applicability.

One limitation of the model is that it focuses solely on 3D‐CRT and IMRT without incorporating alternative treatment modalities such as VMAT or proton therapy (not available).

Another limitation of the model is the variability in predicting the D95% for the PTV across different models. While our models are capable of accurately predicting doses to OARs, there are notable differences in predicting D95% for the PTV, despite the mean dose being well predicted. This discrepancy, although not relevant to the effectiveness of the decision‐making framework, does point to a limitation. We believe that the variability of D95% within the training set between $Inst_{1}$ and $Inst_{2}$ in 3D‐CRT, combined with the variability in lymph node areas (since not all patients have all areas affected), may be contributing to these discrepancies.

We did not evaluate low doses in this study, as these were not part of the routine clinical assessments in the institutions involved. However, in centers where low‐dose evaluation is critical, this could pose a problem. As seen in Figure 3, the IMRT models tend to have higher prediction errors in the low dose regions. This suggests that in institutions where low doses are closely monitored, such as V5Gy, a more careful approach and additional actions may be necessary to ensure that the predictions meet the clinical plans.

Finally, the IMRT patient database, due to clinical workflow, might have a lower number of simple geometries. However, based on the results from the confusion matrix, this issue does not seem to impact the proposed decision‐making framework's ability to correctly decide on the technique.

Moreover, a review of historical decision‐making outcomes (from patients' clinical histories) compared to the proposed decision‐making framework revealed that more IMRT was performed than necessary. This suggests that the selected database likely contained more cases of simple geometries than anticipated.

## 5 CONCLUSIONS

In conclusion, our study introduces a novel decision‐making framework for breast cancer treatment planning utilizing CNN. The developed 2D U‐net model exhibits promising results in predicting dose distributions automatically for both 3D‐CRT and IMRT techniques, especially regarding OAR. The proposed models offer reliable insights into OAR doses (with means around 0% and standard deviation around 1.5% to 5%), facilitating the selection of the most appropriate treatment technique with less bias for individual patients. This decision‐making framework significantly outperforms historical decision‐making, demonstrating higher accuracy, recall, and precision, all exceeding 90%, while optimizing the workflow, saving both time and resources.

## CONFLICT OF INTEREST STATEMENT

The authors declare that they have no known competing financial interests or personal relationships that could have appeared to influence the work reported in this paper.
